# Clinical and epidemiological characteristics of invasive non-typhoidal *Salmonella* disease in Ghana

**DOI:** 10.1371/journal.pone.0362186

**Published:** 2026-11-06

**Authors:** Francis Opoku Agyapong, Michael Owusu, Michael Owusu-Ansah, Sampson Twumasi-Ankrah, Kennedy Gyau Boahen, Gifty Nkrumah, Ezekiel Bonwin Ackah, Rachel Laadi Sulla, Akua Serwaa Boafo, Nathaniel Nsiah-Poku, Portia Okyere Boakye, Anthony Enimil, Yaw Adu-Sarkodie, Ellis Owusu-Dabo, Florian Marks

**Affiliations:** 1 Department of Clinical Microbiology, Kwame Nkrumah University of Science and Technology, Ghana; 2 Department of Medical Diagnostics, Kwame Nkrumah University of Science and Technology, Ghana; 3 Department of Community Health, Kwame Nkrumah University of Science and Technology, Ghana; 4 Department of Statistics and Actuarial Science, Kwame Nkrumah University of Science and Technology, Ghana; 5 School of Public Health, Kwame Nkrumah University of Science and Technology, Kumasi, Ghana; 6 KNUST-IVI Collaborative Centre, Agogo, Ashanti Region, Ghana; 7 Department of Child Health, Kwame Nkrumah University of Science and Technology, Ghana; 8 International Vaccine Institute, Seoul, South Korea; University of Health and Allied Sciences, GHANA

## Abstract

**Background:**

Invasive non-typhoidal *Salmonella* (iNTS) disease remains a significant public health threat in sub-Saharan Africa (SSA). Although the disease is important, its clinical characteristics are not well described in literature. This study describes the clinico-epidemiological profile of iNTS disease in Ghana aiming to bridge important knowledge gaps and inform more effective management strategies.

**Methods:**

The study spanned 8 years and utilized selected fever surveillance sites in Ghana. Patients with fever and/or history of fever for ≥ 3 consecutive days within the week prior to visit were approached and blood sample taken after consenting for investigations including blood culture. iNTS isolates were recovered using BACTEC blood culture, identified by Analytical Profile Index (API), sero-grouped using the Kauffmann–White scheme and confirmed by Illumina whole genome sequencing. Conditional logistic regression was used to assess associations between clinical and laboratory features and iNTS blood culture positivity in a 1:3 age-, sex-, and residence-matched case–control analysis.

**Results:**

Of 271 blood-culture-derived bacterial pathogens, 15.5% (42/271) were identified as iNTS making it the second most common cause of bacteremia. iNTS disease exhibited seasonality with peak detections coinciding with the rainy seasons in Ghana. 23.7% (9/38) of iNTS isolates were multidrug-resistant (MDR), of which 5.3% (2/38) were extensively drug-resistant including resistance to ceftriaxone and ciprofloxacin. Several factors showed crude associations with iNTS disease; however, only anaemia retained its association after adjustment of other factors (aOR=2.41 *p* = 0.030).

**Conclusion:**

iNTS is a major cause of bloodstream infections with the highest burden in children under 5 years of age with susceptibility to the infection amplified by anaemia. The *Salmonella* serovars involved are increasingly becoming multidrug-resistant, including resistance to critical antibiotics.

## Background

The most medically important subspecies of *Salmonella enterica* comprise of over 2500 serovars [1]. These serovars are broadly grouped as typhoidal and non-typhoidal *Salmonella*. The Typhoidal *Salmonella* group has a few members comprising of *S*. Typhi, *S*. Paratyphi A, B and C; all other serovars constitute the non-typhoidal group [2]. Despite the diversity of the non-typhoidal group, *S.* Typhimurium and *S*. Enteritidis together cause over 90% of non-typhoidal *Salmonella* diseases [2].

Non-typhoidal *Salmonellae* (NTS) are important causes of food-borne infections worldwide resulting in acute gastroenteritis [3]. Many routes are responsible for the transmission of NTS to humans including consumption of contaminated food products especially poultry products, undercooked meat, food contaminated with animal waste, consumption of contaminated water and contact with animal reservoirs or their contaminated environment [4,5]. Such infections usually result in self-limited acute gastroenteritis. However, invasive disease (iNTS) – invasion of the bacteria beyond the gut, leading to bacteraemia with or without focal infections which could be fatal without treatment is a well-known occurrence, typically among infants and young children in Sub-Saharan Africa (SSA) and immunocompromised patients of all ages [6–8]. In contrast, in developed countries, NTS usually causes self-limiting gastroenteritis in healthy individuals without posing major health threats [9]. Invasive NTS (iNTS) is usually not associated with gastroenteritis but presents with non-specific features indistinguishable from other common febrile illnesses [6].

The burden of iNTS is huge. Globally, overall incidence rate of 7.5 per 100,000 person-years has been documented [10]. An overwhelming proportion of the global incidence of iNTS disease occurs in SSA, accounting for 80% of global iNTS burden with an incidence rate of about 34.5 per 100,000 person-years with approximately 4100 deaths per year occurring mainly in infants and young children [3,10,11]. The recent Global Burden of Disease estimated the death rate of iNTS to be 76.7 per 100,000 [12]. Rural Ghana has been noted for one of the highest incidences of iNTS disease [13].

Diagnosis of iNTS is established by means of microbiological culture isolating the *Salmonella* organism. Blood culture is usually employed despite its limitation of low sensitivity. Currently there is no rapid diagnostic test for iNTS disease; the commercially available rapid anti-body tests and the obsolete Widal tests are not applicable to iNTS disease diagnostics [14,15].

Despite the huge burden of iNTS disease, the disease is under-recognised, and many clinicians are still not familiar with the disease even in SSA where iNTS is most concentrated [16]. This under-recognition may partly be due to the non-specific clinical presentation of iNTS disease and the difficulty with establishing diagnosis as there are no rapid diagnostic tests and microbiological culture is not routinely performed in most health facilities in the region. Invasive NTS disease burden is further compounded by emerging strains that are multi-drug-resistant leading to a resort to more expensive alternative anti-biotics [17].

Environmental and seasonal factors may influence the epidemiology of iNTS disease. Seasonal variation in iNTS has been reported in sub-Saharan Africa, with rainfall implicated as a potential determinant [18]. In Ghana, seasonality of *Salmonella* bloodstream infections coinciding with rainfall has been reported, although the pattern was more apparent for typhoidal *Salmonella* than for non-typhoidal *Salmonella* [19].

Several studies have described malnutrition [20–22], anaemia, acute severe malaria, recent malaria [8], sickle cell anaemia and other haemoglobinopathies [23] to be associated with iNTS. Rural Ghana has been described as one of the regions with a high burden of iNTS disease [24,25].

Despite these reports, the clinical spectrum and predisposing factors to iNTS disease are not fully understood in Ghana and the West African sub-region. There is still a significant knowledge gap in the antimicrobial resistance (AMR) profiles of iNTS isolates to guide treatment strategies and public health planning in Ghana and many developing countries. The study aimed to characterize the clinical, epidemiological, and antimicrobial resistance profiles of iNTS in Ghana, including temporal and seasonal patterns and their association with rainfall.

## Methods

### Study design and setting

The study was retrospective hospital-based matched case – control analysis utilising data from 01/05/2016–31/12/2023 from fever surveillance studies – *The Severe Typhoid in Africa* (SETA PLUS) and its antecedent study *Surveillance of Enteric fever in Africa* (SETA). SETA PLUS (01/02/2020–31/12/2023) and its forerunning SETA study (26/05/2016–31/01/2020) have been fever surveillance studies aimed at investigating the aetiology of febrile illness in patients reporting with fever and/or history of fever in Ashanti Region of Ghana. SETA PLUS study is on-going in Ghana, Democratic Republic of Congo, Burkina Faso, Madagascar and Nigeria [26]. In Ghana, the fever surveillance sites comprise of four hospitals including two urban hospitals in the Kumasi Metropolis in the Ashanti Region; Komfo Anokye Teaching Hospital – KATH (which is a tertiary facility and the second largest hospital in Ghana) and Maternal and Child Health Hospital (MCH). The other two hospitals considered rural facilities are the Agogo Presbyterian Hospital (APH) located in the Asante Akim North Municipality and Konongo Government Hospital (KGH) in the Asante Akim Central Municipality both in the Ashanti Region (Fig 1).

**Fig 1:**
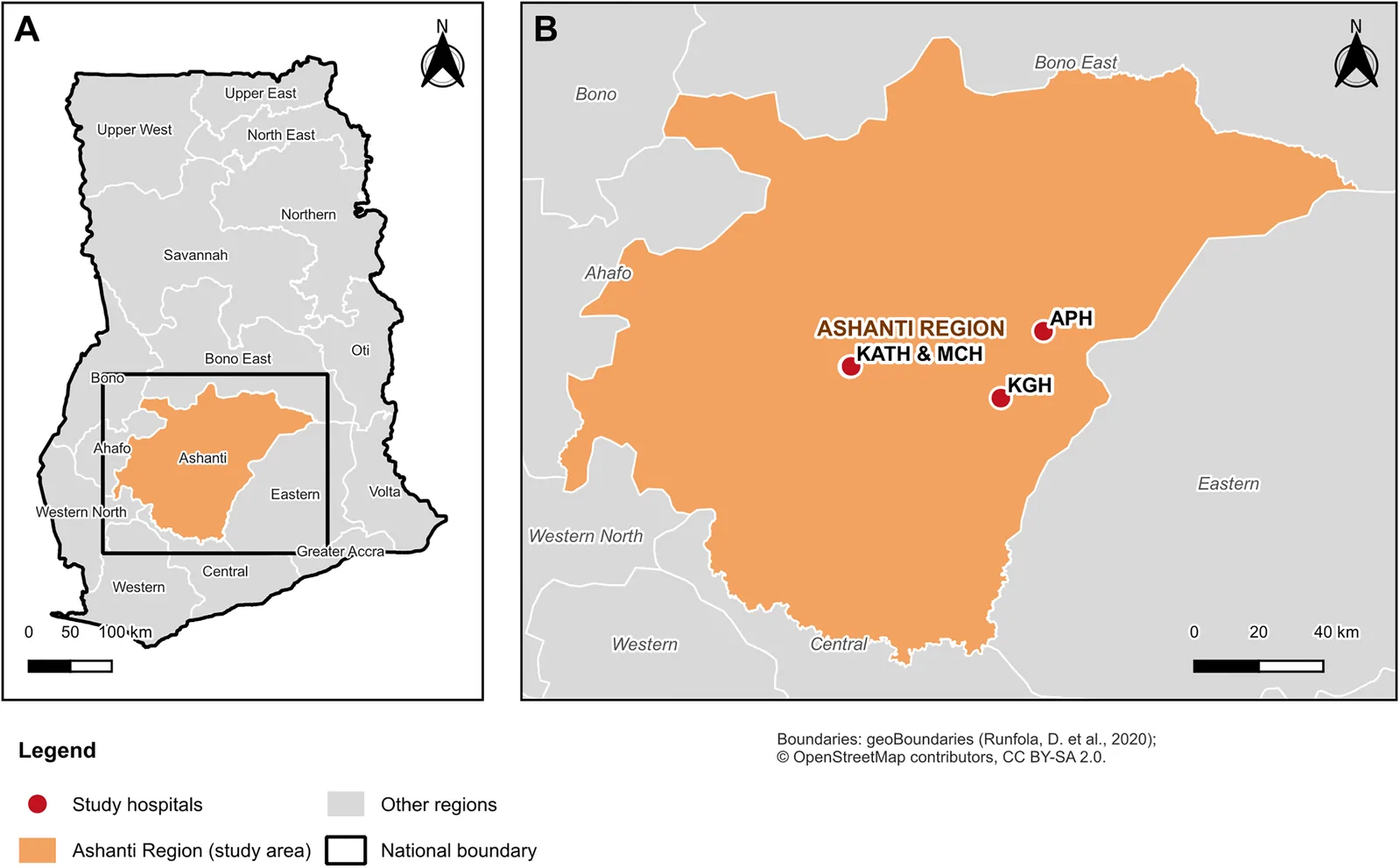
Map Showing Sampling Sites.

### Sample and processing

Clinical data and blood culture samples were collected from patients who reported to the surveillance sites in Ghana with current fever (axillary temperature ≥38°C) and/or a history of fever ≥3 consecutive days within the past 7 days and gave consent. Similarly, blood was also taken for blood film microscopy for malaria parasite detection, haematological and biochemical analytes analyses. Samples were conveyed to the laboratory and processed within two (2) hours of collection.

Blood specimens were aseptically inoculated into BACTEC™ Adult and Peds Plus™/F blood culture bottles (Becton Dickinson, USA) and loaded into the BACTEC automated blood culture system within 2 hours of collection. Positive cultures were subsequently subcultured onto blood agar, chocolate agar, and MacConkey agar for bacterial isolation. Presumptive *Salmonella* isolates were identified based on Gram stain morphology and characteristic growth patterns on culture media. Further biochemical characterization was performed using Triple Sugar Iron, urease, oxidase, and citrate utilization tests, together with the Analytical Profile Index 20E (bioMérieux, France). Serogrouping was carried out using BD Difco™ *Salmonella* antisera (Becton Dickinson, USA) according to the Kauffmann–White classification scheme to distinguish invasive non-typhoidal *Salmonella* from *Salmonella* Typhi (Fig 2).

**Fig 2:**
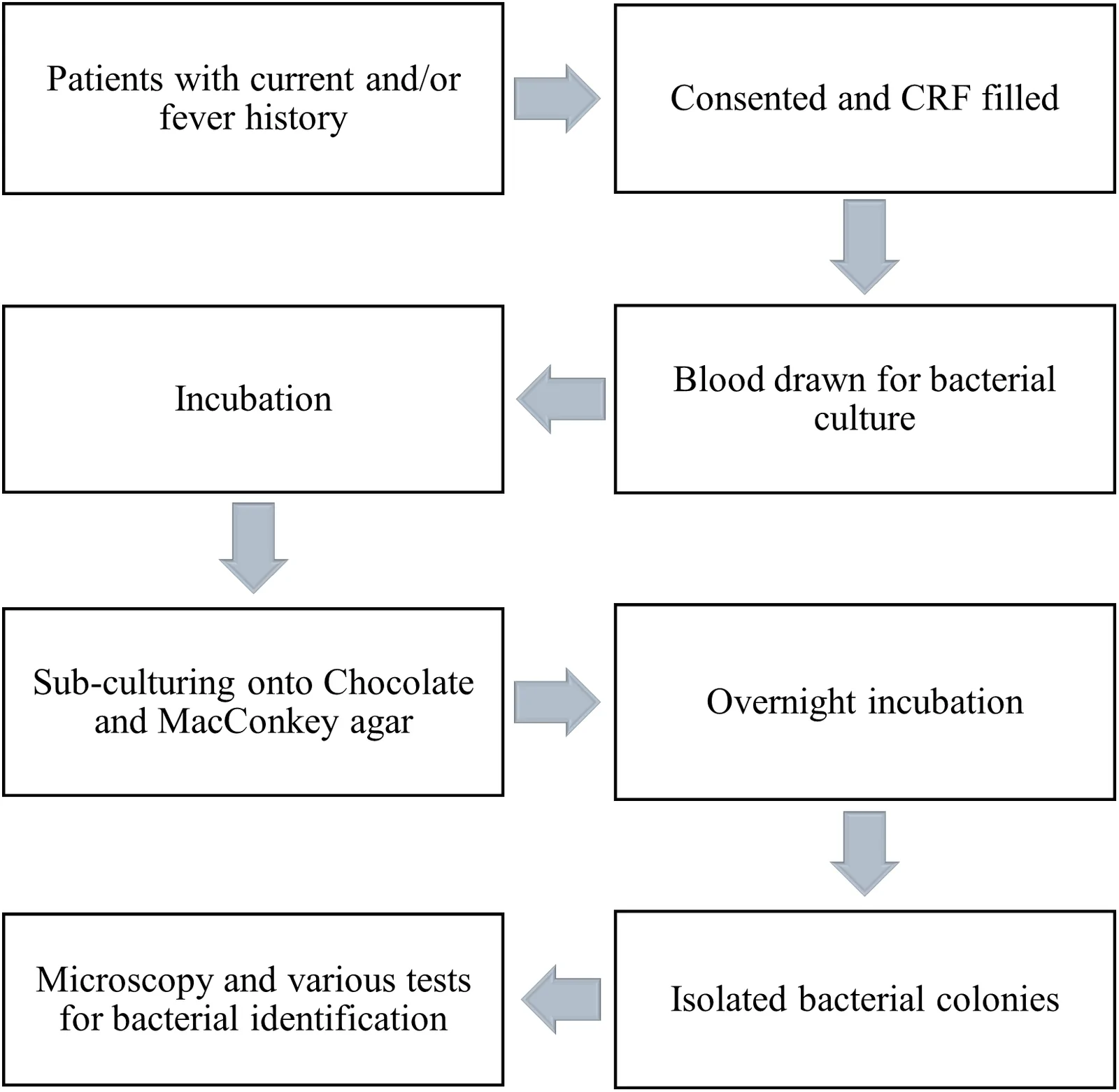
Study flowchart – This flowchart illustrates the workflow for microbiological analysis, beginning with sample collection from consented patients with current fever and/or history of fever for 3 days in the week. This process involves inoculating a culture medium, followed by incubation to allow bacterial growth. After incubation, various media are utilized to isolate and identify bacterial colonies. The final stage demonstrates the use of biochemical tests and other identifying tests to characterize Non-Typhoidal *Salmonella*.

Antimicrobial susceptibility testing was performed on the *Salmonella* isolates using a panel guided by the organism-specific recommendations of the Clinical and Laboratory Standards Institute (CLSI) applicable during the respective years of surveillance. The panel comprised ampicillin (10 µg), chloramphenicol (30 µg), cotrimoxazole (25 µg), amoxicillin-clavulanic acid (20/10 µg), tetracycline (30 µg), ciprofloxacin (5 µg), and ceftriaxone or cefotaxime (30 µg). The panel included CLSI-recommended agents for Salmonella, including ampicillin, a fluoroquinolone, trimethoprim-sulfamethoxazole, and third-generation cephalosporins for extraintestinal isolates, together with additional agents to provide a broader characterization of antimicrobial resistance. Susceptibility testing and interpretation were performed according to the CLSI standards applicable during the respective years of testing. For analysis, isolates classified as intermediate or resistant were considered not susceptible. Confirmed iNTS isolates were preserved at −80°C for downstream DNA extraction and whole-genome sequencing analyses.

Cryopreserved iNTS isolates were revived by subculturing on nutrient agar for recovery. Genomic DNA was subsequently extracted and purified from recovered isolates using the Epicentre Biotechnologies DNA extraction kit (USA) according to the manufacturer’s protocol. DNA concentration was quantified using the Qubit™ dsDNA BR assay kit on the Qubit™ Fluorometer (Thermo Fisher Scientific, USA), and only samples meeting the minimum input threshold of ≥50 ng total DNA were advanced for sequencing.

Whole-genome sequencing was conducted by Eurofins Genomics (Konstanz, Germany) on the Illumina NovaSeq X platform using paired-end 150-bp chemistry. In brief, genomic DNA was processed through fragmentation, end-repair, adenylation, adapter ligation, size selection, and amplification. Libraries were subsequently subjected to quality assessment, with target fragment sizes ranging from 300–600 bp, before pooling and sequencing in PE150 mode to generate paired-end reads and *Salmonella* serovar was determined *in silico* for the isolates using the *Salmonella* *in silico* typing resource (SISTR) [27] tool in the usegalaxy.eu platform [28].

### Data collection and management

Consented participants’ information including demographic, clinical history, physical examination findings, laboratory investigations (including full blood count, liver function test, kidney function test, blood film microscopy and blood culture results), diagnosis and treatment were documented on paper case report forms (pCRF) and entered in pre-tested and launched RedCap system.

Data on patients with positive blood cultures were extracted from the SETA/SETA PLUS-Ghana RedCap database using a standardized abstraction form. The dataset included both continuous and categorical variables encompassing demographic, clinical, and laboratory characteristics of inpatients and outpatients. Time-related measures, expressed in days, comprised: length of hospitalization, duration of current fever, duration of previous fever episodes, interval between onset of symptoms and first healthcare contact, and duration of illness from symptom onset to completion of blood culture testing. Clinical data included self-reported and elicited symptoms and signs. Laboratory data included haematological panels – complete blood count, blood film for malarial parasite microscopy and sickling/haemoglobin electrophoresis reports. Biochemical laboratory data included liver chemistries and renal function reports. The abstracted data in Microsoft Excel were then imported into STATA (StataCorp, College Station, TX, USA) version 17 for analysis. Statistical significance was defined as a two-tailed *p* < 0.05.

### Data analysis

Cumulative monthly counts of invasive non-typhoidal *Salmonella* (iNTS) cases over the 8-year surveillance period were aggregated according to the month of occurrence. Corresponding monthly total rainfall for the fever surveillance region was obtained from the Ghana Meteorological Services. For each calendar month (January–December), the mean total rainfall was calculated across the 8-year period to generate representative monthly rainfall profiles. Monthly cumulative iNTS case counts were initially assessed for association with average monthly rainfall using Spearman rank-order correlation as an exploratory non-parametric analysis. To more robustly evaluate the temporal relationship while accounting for autocorrelation and potential non-linear effects, a generalized additive model with autoregressive terms was subsequently fitted. The effective degrees of freedom (edf), reference degrees of freedom (ref.df), F-statistic, and corresponding p-values were used to assess the fitted smooth term.

To assess the associated factors with iNTS disease, conditional logistic analyses were performed to ascertain characteristic clinical and laboratory features if any among the iNTS cases compared to blood culture-negative patients by using 105 blood culture–negative controls matched to iNTS-positive cases by age, sex, and area of residence yielding 1:3 case-control pairs.

Variables with a p-value <0.25 in the univariable analysis were included in a multivariable logistic regression model to identify independent associations of iNTS disease. Adjusted odds ratios (aORs) with corresponding 95% confidence intervals (CIs) were reported. Model diagnostics were conducted to evaluate model fit. Goodness-of-fit was assessed using the Hosmer–Lemeshow test and the area under the ROC curve (AUC).

### Ethics statement

Ethics approval for this study was obtained from the Committee on Human Research Publication and Ethics (CHRPE) of the School of Medical Sciences, Kwame Nkrumah University of Science and Technology (KNUST), Kumasi, Ghana with yearly renewals; (CHRPE/AP/219/16, CHRPE/AP/594/17, CHRPE/AP/188/18, CHRPE/AP/605/19, CHRPE/AP/031/20, CHRPE/AP/071/21, CHRPE/AP/282/22, CHRPE/AP/111/23). Written informed consent was obtained from participants above 18 years who were enrolled in the surveillance study. Child assent and parental/guardian consent were obtained for children less than 18 years. The anonymity and confidentiality of the participants were assured using unique codes. Participants who did not provide consent were excluded from the study.

## Results

### Demography and blood culture outcome of patients enrolled

Two fever surveillance sites involving APH in Agogo and KATH, Kumasi were involved in the SETA fever surveillance study. The surveillance sites expanded to include MCH in Kumasi and KGH in Konongo during the SETA PLUS phase. Over the 8-year study period, a total of 14,824 enrolled patients received blood culture (Table 1). Females 7622 (51.4%) constituted majority of patients and the under 5-year group had the greatest number of enrollees 5612 (37.9%) with teenagers (13–19 years) constituting the least number of recruited patients 1756 (11.8%) (Table 1).

**Table 1:** Demography of patients who received blood culture.

Gender	Frequency (%)	95% CI
Males	7202 (48.6%)	47.8% − 49.4%
Females	7622 (51.4%)	50.6% − 52.2%
Total	14824 (100.0%)	
**Age Groups**		
< 5 years	5612 (37.9%)	37.1% − 38.6%
≥5 years	9212 (62.1%)	61.4% − 62.9%
Total	14824 (100.0%)	

Data are presented as frequency (percentage), with corresponding 95% confidence intervals (CIs).

A total of 271 (1.8%) of the blood cultures were positive for clinically significant bacteria. Among the clinically significant bacteria, *Salmonella* isolates constituted about half (50.6%, 137/271) with *Salmonella* Typhi accounting for 35.1% (95/271) and iNTS organisms constituting 15.5% (42/271) of all the blood culture confirmed bacteremia cases (Table 2). This made iNTS organisms the second most common cause of bacteremia among the patients. Other predominant organisms in order of decreasing frequency are shown in Table 2. Of the iNTS organisms sequenced and serotyped *in-silico*, 67.7% yielded the serotyped iNTS isolates as principal serovars – *S*. Typhimurium and *S*. Enteritidis while the remaining 33.7% comprised of non-principal serovars (i.e., non-Enteritidis and non-Typhimurium) including *S*. Saintpaul, *S*. Poona. *S.* Vichow, *S*. Matadi, *S*. Stanleyville, *S.* Ajiobo*, S.* Waycross and *S.* Wien.

**Table 2:** Blood-borne pathogens from the patients.

Isolate	Frequency (%)
*Salmonella* Typhi	95 (35.1%)
Invasive Non*-*Typhoidal *Salmonella*	42 (15.5%)
*Escherichia coli*	23 (8.5%)
*Staphylococcus aureus*	23 (8.5%)
*Pseudomonas* spp	15 (5.5%)
*Streptococcus pneumonia*	14 (5.2%)
*Acinetobacter* spp	11 (4.1%)
*Klebsiella* spp	10 (3.7%)
*Burkholderia* spp	5 (1.8%)
*Enterobacter cloacae*	4 (1.5%)
*Hemophilus influenzae*	3 (1.1%)
*Enterococcus* spp	2 (0.7%)
Group C *streptococci*	2 (0.7%)
*Neisseria* spp	2 (0.7%)
*Ochrobactrum anthropi*	2 (0.7%)
*Pasteurella pneumotropica*	2 (0.7%)
*Sphingomonas paucimobilis*	2 (0.7%)
*Alcaligenes faecalis*	1 (0.4%)
*Chromobacterum violaceum*	1 (0.4%)
*Chryseobacterium indologene*	1 (0.4%)
*Citrobacter koseri/fermeri*	1 (0.4%)
*Group A streptococci*	1 (0.4%)
*Listeria* spp	1 (0.4%)
*Morexella catarrhalis*	1 (0.4%)
*Pantoea* spp	1 (0.4%)
*Ralstonia picketti*	1 (0.4%)
*Raoultella ornithinolytica*	1 (0.4%)
*Streptococcus* spp	1 (0.4%)
*Vibrio alginolyticus*	1 (0.4%)
*Weeksella virosa*	1 (0.4%)
Gram Negative Rod (unclassified)	1 (0.4%)
**Total**	(100.0%)

Data are presented as frequency (percentage). Percentages are based on the total number of bacterial isolates recovered (n = 271). spp – species.

### Analysis of the demography of blood culture positive patients

Of the 271 patients with positive cultures, 45.4% (122/271) were females, 64.9% (176/271) were rural dwellers and the under-5-year-old group constituted 42.8% (116/271). Pathogen distribution differed significantly by both age group and study area. By age, iNTS was proportionally more common among children aged <5 years than among patients >5 years (25.0% vs 8.4%), whereas *S*. Typhi was more frequently detected in patients >5 years (49.0% vs 16.4%) (p < 0.001). Similarly, significant variation was observed across study areas (p < 0.001), with *S*. Typhi occurring more frequently among rural participants than urban participants (43.2% vs 20.0%), while other pathogens predominated in urban settings (65.3% vs 40.9%). In contrast, the proportion of iNTS was comparable between urban and rural settings (14.7% vs 15.9%) (Table 3).

**Table 3:** Demography of blood culture-positive patients (n = 271).

Gender	iNTS Positive (n=42)	*S*. Typhi Positive(n = 95)	Other Pathogen Positive(n = 134)	P-value
Males	19 (12.8%)	59 (39.6%)	71 (47.7%)	0.151
Females	23 (18.9%)	36 (29.5%)	63 (51.6%)	
**Study Area**				**<0.001**
Urban	14 (14.7%)	19 (20.0%)	62 (65.3%)	
Rural	28 (15.9%)	76 (43.2%)	72 (40.9%)	
**Age Group**				**<0.001**
<5 years	29 (25.0%)	19 (16.4%)	68 (58.6%)	
≥5 years	13 (8.4%) (9.5%)	76 (49.0%)	66 (42.6%)	

Data are presented as frequency (percentage). Percentages are calculated within each demographic or study-area category*. P*-values were derived using Pearson’s chi-square test. iNTS, invasive non-typhoidal Salmonella.

### Clinical characteristics of the iNTS patients

A total of 35 including 7 iNTS blood culture positive patients had incomplete enrolment data beyond the demography. Therefore 7 iNTS cases were excluded from the iNTS-predisposing factors and clinical features analysis (Fig 3).

**Fig 3:**
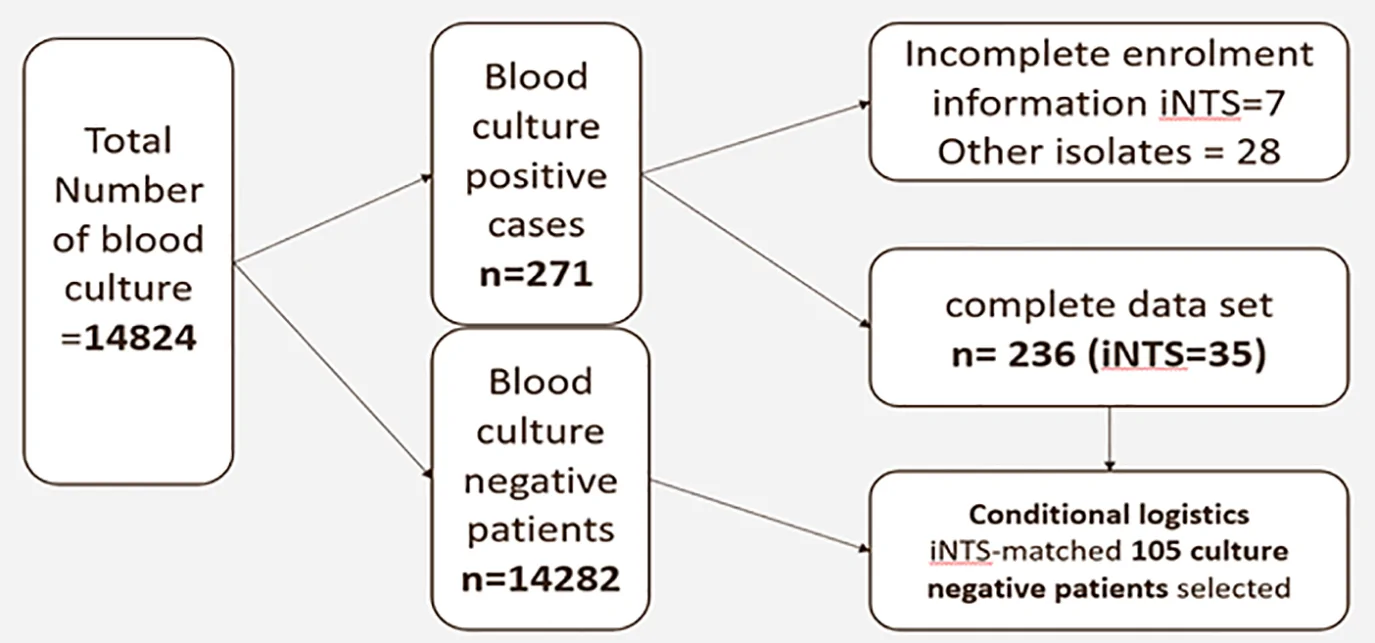
Flowchart of figures used in the statistical analysis.

Among the 35 positive iNTS cases with complete enrolment information (Fig 3), variable clinical features were reported. Eighty percent (28/35) were febrile at the point of enrolment; the commonest presenting symptom was cough (48.6%; 17/35) and the least common presenting complaints were seizure (5.7%; 2/35) and dyspnoea (5.7%; 2/35). Antibiotic intake for their current illness before blood draw for culture was reported among 14.3% (5/35) of the iNTS cases. Pre-existing comorbidity including sickle cell disease/anaemia, cardio-renal disease and HIV infection was reported among 40% (14/34) of the iNTS cases. Laboratory analyses revealed 88.6% (31/35) of the iNTS cases as anaemic, and 88.6% (31/35), 71.4% (25/35) and 65.7% (23/35) had low haematocrit, low red cell count and raised creatinine levels respectively based on WHO age-gender-based definition/categorization of these analytes [29]. Of the 35 iNTS cases, 57.1% (20/35) were managed as in-patients and none of the blood-culture confirmed iNTS cases was initially managed as or suspected of having *Salmonella*-related disease before the blood-culture report.

### Associated factors with iNTS disease

Assessment of factors associated with iNTS disease was made using conditional logistic regression analysis among 140 patients comprising of the 35 iNTS cases and their 1:3 age-gender-place of residence matched blood culture negative controls yielding 1:3 case-control pairs (table 4).

**Table 4:** Numbers used in the case:control analysis (105 age-gender-place of stay matched controls to the iNTS cases were randomly selected for conditional logistic regression.

Gender iNTS cases	Culture-negative control	Frequency (%)
Males 15	45	60 (42.9%)
Females 20	60	80 (57.1%)
**Study Area**		
Urban 12	36	48 (34.3%)
Rural 23	69	92 (65.7%)
**Age Group**		
<5 years 25	75	100 (71.4%)
≥5 years 10	30	40 (28.6%)

Each iNTS case was matched to three culture-negative controls by sex, age group, and study area. Data are presented as the number of iNTS cases and corresponding controls, with combined frequencies and percentages shown in the final column. iNTS, invasive non-typhoidal Salmonella.

Compared to the matched blood-culture negative patients, jaundiced patients and those with splenomegaly had significantly increased odds of iNTS detection (cOR=7.5; *p* = 0.016 and cOR=12; *p* = 0.026 respectively) (Table 5). Similarly, anaemia (cOR=4.71; *p* = 0.005), reduced red blood cell count (cOR=11.38; *P* < 0.001), low haematocrit (cOR=4.9; *P* = 0.005), and elevated creatinine levels (cOR=19.35; *P* < 0.001) were significantly associated with higher odds of iNTS compared with blood culture–negative patients. Further, being managed as inpatient (cOR = 9.46; *P* < 0.001,) and having underlying comorbidity (cOR=24.97; *P* = 0.002) were significantly associated with iNTS disease. However, in the multivariate logistic model, when adjusted for other factors, the significance of these factors’ association with iNTS disease was lost except for anaemia and the odds of inpatient management, which retained their significance (Table 5). This makes anaemia an independent predisposition factor to iNTS disease with the tendency of being admitted for in-patient treatment being associated with increased odds of iNTS disease among the study population. Although not statistically significant, raised white cell count and neutrophilia appear to be a negative predictor of iNTS disease with the multivariate logistic analysis (Table 5).

**Table 5:** Conditional logistic regression analysis of clinical characteristics of iNTS-positive patients versus matched blood culture–negative controls (1:3 case–control matching by age, sex, and place of residence).

Symptoms/Signs	iNTS Positive (n=35)	Controls (n = 105)	cOR	*P*-value	aOR	*P*-value	95% CI
Presence of Current fever	28 (25.4%)	82 (74.6%)	1.13	0.808	–	–	–
Having Cough	17 (32.1%)	36 (67.9%)	1.90	0.120	2.49	0.286	0.49–10.54
Abdominal pains present	5 (23.8%)	16 (76.2%)	0.93	0.893	–	–	–
Having Diarrhoea	6 (40.0%)	9 (60.0%)	2.08	0.179	7.43	0.055	0.99 - 50.86
Presence of Vomiting	9 (27.3%)	24 (72.7%)	1.16	0.733	–	–	–
Having Headache	7 (31.8%)	15 (68.2%)	1.53	0.417	–	–	–
Experienced Seizure	2 (33.3%)	4 (66.7%)	1.59	0.620	–	–	–
Feeling Malaise	4 (22.2%)	14 (77.8%)	0.84	0.773	–	–	–
Having Dyspnoea	2 (50.0%)	2 (50.0%)	3.00	0.272	–	–	–
Jaundice present	5 (71.4%)	2 (28.6%)	7.50	0.016	4.60	0.85	0.14–47.12
Hepatomegaly present	4 (50.0%)	4 (50.0%)	3.48	0.107	0.21	0.419	0.01–9.23
Splenomegaly present	4 (80.0%)	1 (20.0%)	12.00	0.026	2.24	0.682	0.04 −106.80
Prostration present	3 (75.0%)	1 (25.0%)	9.00	0.057	–	–	–
Having sore throat	1(33.33%)	2 (66.67%)	1.5	0.741	–	–	–
**Laboratory features**							
Having anaemia	31(33.3%)	62(66.7%)	4.71	0.005	2.41	0.030	0.82–7.8
Malaria parasitemia	16(30.8%)	36 (69.2%)	1.63	0.229	1.10	0.184	0.05–1.77
Raised white cell count	10 (38.5%)	16(61.5%)	2.45	0.073	0.71	0.759	0.08–6.08
Low red cell count	25 (52.1%)	23 (47.9%)	11.38	<0.001	3.64	0.281	0.35–38.27
Low haematocrit	31 (33.0%)	63 (67.0%)	4.90	0.005	4.54	0.341	0.28–28.46
Neutrophilia	12 (19.3%)	50 (80.7%)	0.54	0.157	0.389	0.249	0.08–1.93
Eosinophilia	4 (57.1%)	3 (42.9%)	4.00	0.070	5.74	0.057	0.10–98.23
Raised serum creatinine	23 (69.7%)	10 (30.3%)	19.35	<0.001	–	–	–
**Other clinical features**							
Prior antibiotic intake	5 (45.5%)	6 (54.55%)	2.71	0.120	1.24	0.857	0.12–13.34
Presence of comorbidity	14 (50.0%)	14 (50.0%)	24.97	0.002	11.83	0.144	0.19–44.10
Managed as in-patient	20 (47.6%)	22 (52.4%)	9.46	<0.001	4.09	0.045	0.59–28.47

Data are presented as frequency (percentage). cOR, crude odds ratio; aOR, adjusted odds ratio; CI, confidence interval. Odds ratios and 95% CIs were estimated using conditional logistic regression. NB. Prostration and serum creatinine were excluded from the multivariable logistic regression model due to model non-convergence, likely related to sparse data and unstable parameter estimates; comorbidities (HIV, Sickle cell disease/anaemia, G6PD Deficiency, malnutrition, cardiac or renal disease) were not analysed individually because sparse data produced unstable parameter estimates.

The multivariable logistic regression model demonstrated adequate fit (Hosmer–Lemeshow *p* = 0.34; AUC = 0.79).

### Seasonal distribution of iNTS cases

Exploratory Spearman rank-order correlation analysis demonstrated a moderate positive association between cumulative monthly iNTS cases and average monthly rainfall (r_s_ = 0.63, p = 0.030; n = 12;). However, when temporal dependence was accounted for using a generalized additive model with autoregressive terms (GAMAR), the relationship was approximately linear (edf = 1, ref.df = 1) and showed a positive trend though not statistically significant (F = 4.658, p = 0.0563; Fig 4, S1 Fig).

**Fig 4:**
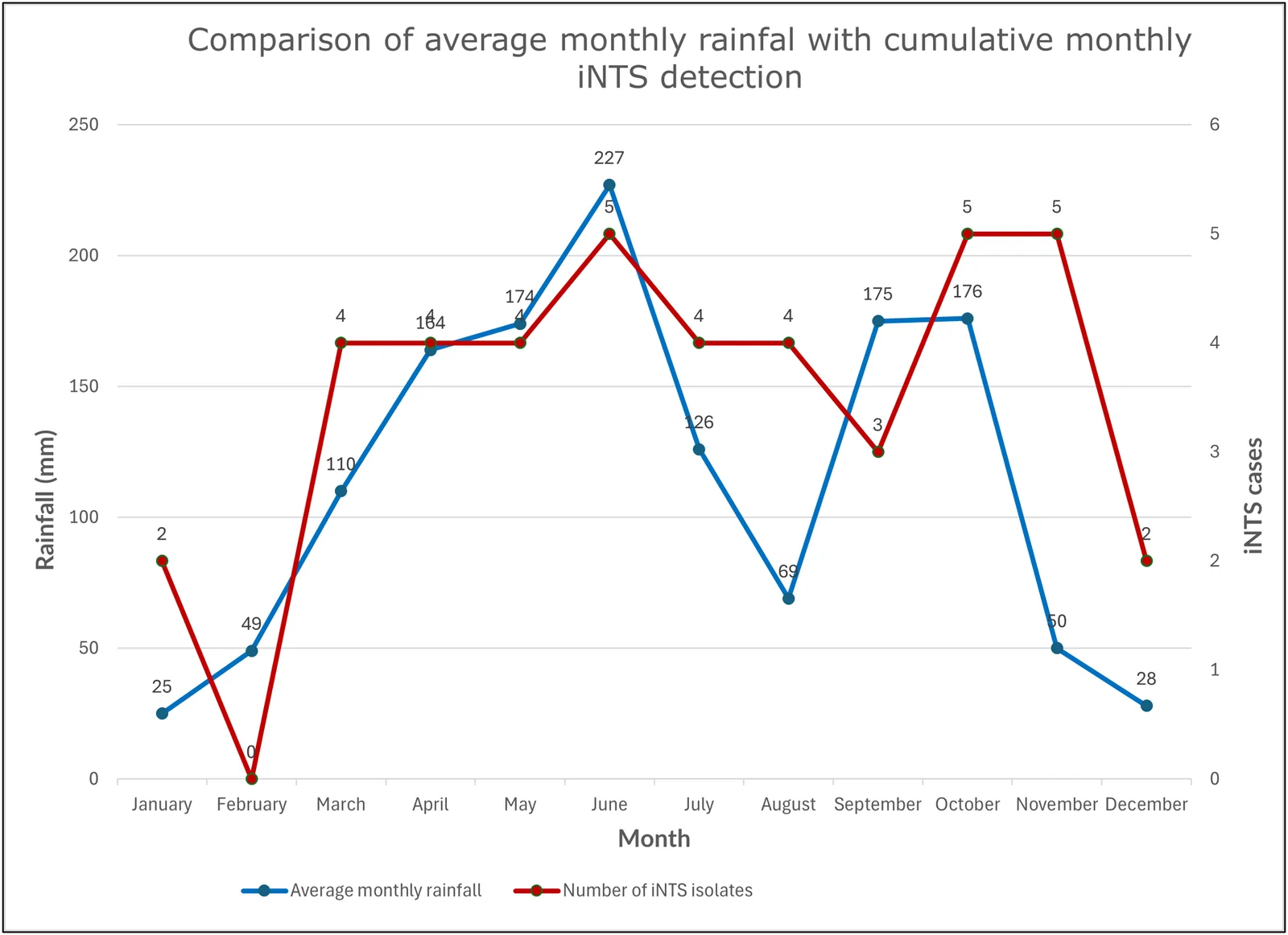
Monthly distribution of invasive non-typhoidal *Salmonella* (iNTS) cases and mean total rainfall (measured in millimetres at the study areas over an 8-year surveillance period.

### Antimicrobial susceptibility patterns of iNTS isolates

Invasive NTS recorded the highest resistance to ampicillin 34% (13/38). Thirty-One percent (31%; 12/39) of the isolates were not susceptible to ciprofloxacin (2/39 with overt resistance and 10/39 with intermediate resistance). For the third-generation cephalosporin – Ceftriaxone or cefotaxime, 14% (5/37) of the iNTS isolates were not susceptible (2/37 showed complete resistance while 3/37 were intermediate). For 38 iNTS isolates which underwent phenotypic testing against Ampicillin, chloramphenicol and cotrimoxazole, 23.7% (9/38) were found to be multidrug resistant (resistant to ampicillin, chloramphenicol and cotrimoxazole) of which 2 were additionally resistant to ciprofloxacin and ceftriaxone or cefotaxime (Table 6 and S1 Table).

**Table 6:** Anti-microbial resistant patterns of iNTS isolates.

Drug	Resistance	Intermediate	Sensitive
Ampicillin (n = 38)	13/38	3/38	22/38
Chloramphenicol(n = 38)	11/38	–	27/38
Cotrimoxazole (n = 33)	10/33	1/33	22/33
Amoxicillin-Clavulanic acid (n = 35)	3/35	3/35	29/35
Tetracycline (n = 38)	6/38	–	32/38
Ciprofloxacin (n = 39)	2/39	10/39	27/39
Ceftriaxone/Cefotaxime (n = 37)	2/37	3/37	32/37

Values are presented as the number of isolates resistant, intermediate, or sensitive to each antimicrobial agent (n/N).

## Discussion

The study aimed to determine the clinico-epidemiologic characteristics of iNTS disease in the study population.

This study reports iNTS as the second most common aetiology of acute febrile illness accounting for 15.4% of all causes of blood culture confirmed bacteremia. Multiple country-level reviews from SSA consistently demonstrate that iNTS constitutes between 8% and 45% of all bacterial pathogens isolated from blood cultures [6,24], a result which is in keeping with the findings of this study. The actual prevalence rate is likely higher than this figure considering the varying sensitivity of blood cultures (30–80%). Prior antibiotic exposure before blood culture collection may reduce the sensitivity of blood culture for detection of iNTS [4,30–32]. Prior antibiotic intake was assessed in this study and included in the multivariable analysis; however, information on the duration of use, and source of antibiotics was not collected. Other health-seeking behaviours, including the use of herbal remedies and non-attendance at formal health facilities, were also not assessed. These unmeasured aspects of antibiotic exposure and health-seeking behaviour may have influenced the detection of iNTS and should be considered in future investigations.

There is nearly even distribution of iNTS positivity rates between males and females with no association with a particular gender. In the global burden of iNTS disease estimates for 2017, the incidence of iNTS disease was similarly not found to be different statistically between males and females [10,12] and so was the finding from a recent study in Asia [33]. This suggests no gender predisposition for iNTS disease.

This study reports that about 69.0% of all iNTS cases occurred in under 5-year-old children. This finding is consistent with reports by many authors describing iNTS as predominantly early childhood disease [33,34]. However, some researchers describe bimodal age distribution of iNTS disease comprising of under-5 years and third-fourth decades of life [16,35]. Findings from this study invariably conform to many existing studies highlighting the epidemiology of iNTS as mainly a paediatric disease with under-5 years predominantly affected [24,36]. The skewed epidemiology of iNTS disease towards the under 5-year-olds may reflect the general immature immune system, high prevalence of malnutrition and other risk factors such as anaemia possibly from recent or recurrent malaria illness, parasitic infection, haemoglobinopathies etc, high likelihood of exposure to food and water contaminated with environmental materials that may be a source for *Salmonella* among this age group particularly in SSA where iNTS disease is endemic [37–39]. This skewed epidemiology also presents the opportunity of targeted preventive interventions including good WaSH (water, sanitation and hygiene) practices and vaccination. Seasonal trends in iNTS diseases with higher cases during the rainy season has been described in SSA [18,40]. This seasonal pattern in favour of the wet season may be due to heavy rains causing flooding and possible spread of *Salmonella* or increased in malaria cases during the rainy seasons as recent and current malaria have been shown to be associated with iNTS disease in other regions [34,40]. Initial correlation analysis of this study’s data suggested a significant positive association between rainfall and iNTS incidence. However, this association was attenuated after accounting for temporal autocorrelation using generalized additive modelling. This suggests that while rainfall may influence iNTS occurrence, the observed association should be interpreted cautiously. The limited number of monthly observations in this study likely reduced statistical power to detect a robust temporal effect.

Anaemia was found to be associated with iNTS disease by this study. This finding is in keeping with a study in African children which reports severe anaemia as a risk factor as it increases susceptibility to iNTS disease [41]. The disappearance of the association between low haematocrit and reduced red cell count after adjustment may indicate that their effect is mediated through closely related variables such as anaemia which retained its association with iNTS disease in the multivariable analysis.

iNTS disease is generally described as having diverse and non-specific clinical symptoms [10] though some researchers describe it as being associated with fever [13,34] and respiratory symptoms of cough and shortness of breath [42]. This study reveals iNTS in the Ghanaian communities presents as non-specific disease as none of the patients’ presenting symptom/sign was found to be independently associated with the disease in the multivariate regression model. Consequently, due to this non-specificity, none of the culture confirmed iNTS disease cases had initial differential diagnoses of *Salmonella*-related disease and thus were not managed as *Salmonella*-related illness until the blood culture result was available. This diversity and non-specificity in iNTS clinical symptoms portend a major diagnostic blind spot posing challenges to clinical diagnosis. This calls for increased awareness of the disease. Despite the highly diverse and non-specific clinical symptoms and signs, this study shows patients with iNTS disease are more likely to be managed as in-patients than as out-patients compared to blood-culture negative patients, a finding also described in Malawi [42]. This suggests that patients who may be iNTS blood culture positive are likely ‘sicker’ than patients who may be blood culture negative.

Invasive NTS was found to be associated with jaundice and splenomegaly in the univariate analysis, a finding which contrasts a report from Malawi [42]. A closer analysis of the current data shows jaundice and splenomegaly were elicited in sickle cell disease/anaemic patient enrollees reflecting the underlying comorbidity which may be a predisposition factor for iNTS disease rather these signs being direct effect of iNTS disease.

Multidrug resistance was detected among 23.7% (9/38) of the isolates and the most frequent resistance patterns were observed against ampicillin, cotrimoxazole, and tetracycline, consistent with previously reported trends of AMR pattern in iNTS isolates across sub-Saharan Africa [4,34,42,43]. The detection of multidrug resistance among the isolates, including overt or intermediate resistance to ciprofloxacin and ceftriaxone/cefotaxime, is concerning given the importance of these agents for the treatment of invasive *Salmonella* infections. These antimicrobials remain among the most used first-line agents, which may contribute to the sustained selective pressure driving resistance. The high prevalence of MDR and XDR strains underscores the growing threat of limited therapeutic options for iNTS infections. This pattern aligns with findings from several regional and global studies highlighting the persistence of resistance to these legacy antibiotics in clinically important non-typhoidal as well as typhoidal serovars [43].

This study faced limitations on field recruitment activities during the year 2019, this negatively impacted on the estimation of the true burden of iNTS disease and its temporal transmission patterns. Following 2019, 2020 and 2021 febrile cases recruitment greatly suffered from the restrictions that emanated from COVID-19 disease political and public health management. This might have impacted on the true burden of iNTS estimation and its transmission dynamics in the Ghanaian communities. Also, the study suffers a drawback of sampling sites limited to the central belt of Ghana and relatively small number of iNTS pathogens used in the analyses which may make generalization of the findings across the entire country be treated with caution. Information on previous febrile illness treatment, and recurrent or returning episodes was not systematically captured; therefore, previously treated or recurrent iNTS cases could not be identified. Furthermore, although no bacterial–bacterial co-infections were identified, concurrent malaria infection occurred in some patients with iNTS, potentially confounding the attribution of certain clinical manifestations to iNTS.

## Conclusion

This study highlights invasive non-typhoidal *Salmonella* (iNTS) as the second leading cause of bacterial bloodstream infections in Ghana, and children under five years of age are disproportionately affected, irrespective of gender. Clinically, iNTS disease presented with non-specific features, often evading initial differential diagnosis while anaemia emerged as an important predisposition factor consistent with observations across sub-Saharan Africa.

Almost one-quarter of iNTS isolates were multidrug-resistant, with some exhibiting resistance to fluoroquinolones and third-generation cephalosporins, posing a significant treatment challenge. These findings underscore the urgent need for strengthened surveillance, rapid diagnostics, genomic analysis, and targeted interventions to reduce the burden and improve the management of iNTS in Ghana.

## Supporting information

S1 Fig GAMAR plot. Smooth effect of rainfall on iNTS cases.
(PNG)

S1 Table Isolate-level phenotypic antimicrobial resistance patterns of iNTS isolates. (DOCX)
