# Supplementary material for: Clinical and epidemiological characteristics of invasive non-typhoidal Salmonella disease in Ghana
Source: PLoS One. 2026 Nov 6;21(11):e0362186. doi: 10.1371/journal.pone.0362186 (PMC13743023; doi:10.1371/journal.pone.0362186)
Supplement: Supplementary file 1 [file pone.0362186.s002.docx]

**Isolate-level Phenotypic Antimicrobial Resistance Patterns of iNTS Isolates**

| **SN** | **Amoxicillin/Clavulanic acid** | **Ampicillin** | **Ceftriaxone/Cefotaxime** | **Chloramphenicol** | **Ciprofloxacin** | **Cotrimoxazole** | **Tetracycline** |
| --- | --- | --- | --- | --- | --- | --- | --- |
| **1** | S | S |  | S | S |  | S |
| **2** | I | I | I | S | I |  | S |
| **3** | S |  |  | S | S |  | S |
| **4** | S | S | S | S | I |  | S |
| **5** | S | S | I | S | S | S | S |
| **6** | S | S | S | S | I | S | S |
| **7** | S | S | S | S | S | S | S |
| **8** | S | S | S | S | S | S | S |
| **9** | I | I | R | S | I | I | S |
| **10** | R | R | S | S | S | R | R |
| **11** | S | S | S | S | S | S | S |
| **12** | S | S | S | S | S |  | R |
| **13** | S | S | I | S | R | S | S |
| **14** | S | S | S | S | S | S | S |
| **15** | S | S | S | S | S | S | S |
| **16** | R | R | S | R | R | R | R |
| **17** | S | S | S | S | S | S | S |
| **18** | S | S | S | S | S | S | S |
| **19** | S | S | S | S | S | S | S |
| **20** | S | S | S | S | S | S | S |
| **21** | S | S | S | S | S | S | S |
| **22** | S | R | S | R | S | R | S |
| **23** | S | S | S | S | S | S | S |
| **24** | S | R | S | R | S | R | S |
| **25** | S | S | S | S | I | S | S |
| **26** | S | R | S | R | S | R | S |
| **27** | R | R | S | R | S | R | S |
| **28** | S | R | S | R | S | R | R |
| **29** | S | S | S | S | S | S | S |
| **30** | S | S | S | S | I | S | S |
| **31** | I | R | S | R | I | R | R |
| **32** | S | R | S | R | S | S | S |
| **33** | S | S | S | S | I | S | S |
| **34** |  | R | S | R | I | R | R |
| **35** | S | I | S | S | I | S | S |
| **36** |  | R | S |  | S | S |  |
| **37** | S | S | S | S | S |  | S |
| **38** |  | R | R | R | S | S | S |
| **39** |  | R | S | R | S | R |  |

**S.N = serial number, Pat ID=Patient/Isolate Identity number, S=sensitive, I=Intermediate, R= Resistance NB: AMR pattern data for 3 isolates were unavailable. Empty cells indicate that antimicrobial susceptibility results were unavailable because testing was not performed or because the results did not meet quality control criteria and could not be interpreted**
